# Beam management optimization for V2V communications based on deep reinforcement learning

**DOI:** 10.1038/s41598-023-47769-3

**Published:** 2023-11-22

**Authors:** Junliang Ye, Xiaohu Ge

**Affiliations:** Huazhong University of Science and Technology, Wuhan, 430074 China

**Keywords:** Electrical and electronic engineering, Information technology

## Abstract

Intelligent connected vehicles have garnered significant attention from both academia and industry in recent years as they form the backbone of intelligent transportation and smart cities. Vehicular networks now exchange a range of mixed information types, including safety, sensing, and multimedia, due to advancements in communication and vehicle technology. Accordingly, performance requirements have also evolved, prioritizing higher spectral efficiencies while maintaining low latency and high communication reliability. To address the trade-off between communication spectral efficiency, delay, and reliability, the 3rd Generation Partnership Project (3GPP) recommends the 5G NR FR2 frequency band (24 GHz to 71 GHz) for vehicle-to-everything communications (V2X) in the Release 17 standard. However, wireless transmissions at such high frequencies pose challenges such as high path loss, signal processing complexity, long pre-access phase, unstable network structure, and fluctuating channel conditions. To overcome these issues, this paper proposes a deep reinforcement learning (DRL)-assisted intelligent beam management method for vehicle-to-vehicle (V2V) communication. By utilizing DRL, the optimal control of beam management (i.e., beam alignment and tracking) is achieved, enabling a trade-off among spectral efficiency, delay, and reliability in complex and fluctuating communication scenarios at the 5G NR FR2 band. Simulation results demonstrate the superiority of our method over the 5G standard-based beam management method in communication delay, and the extended Kalman Filter (EKF)-based beam management method in reliability and spectral efficiency.

## Introduction

Vehicular communications, which connect vehicles, infrastructures, networks, and pedestrians, are facing significant challenges due to the superior performance requirements brought by the development of intelligent transportation systems and smart cities^1,2^. Specifically, future vehicular networks are required to handle a series of critical tasks, including data collection, traffic coordination, autonomous driving, and energy consumption^3,4^. On the contrary, only four dedicated sub-bands are allocated for V2X communication in the 5G NR FR1 frequency band. To solve this issue, 3GPP has recommended the 5G NR FR2 frequency band for V2X in the Release 17 standard. Although higher frequency bands bring more spectrum resources, it is still a daunting task to efficiently utilize these spectrum resources to achieve low latency, high reliability, and high spectral efficiency communication in V2X communications, especially in the V2V communication scenarios^5^, according to the following reasons.

1. High path loss: The transmissions on the 5G NR FR2 band suffer from higher path loss compared with 5G NR FR1, i.e., sub-6GHz. Thus, larger antenna arrays with massive multiple inputs multiple outputs (MIMO) systems and narrower beam patterns are required by both transmitters and receivers to compensate for the high path loss of the wireless links^6^. However, larger antenna arrays lead to higher signal processing complexity. Moreover, the narrow beam patterns result in higher time costs and smaller beam coverage in beam alignment, beam training, and beam tracking^7^. These facts further affect the end-to-end transmission delay and reliability of V2V communications.
2. High mobility: High mobility is a distinctive feature of vehicle-to-vehicle (V2V) communication, where both the transmitter and the receiver are in motion at high velocity. This makes a V2V communication channel more unpredictable, variable, and unstable in comparison to vehicle-to-infrastructure (V2I) and vehicle-to-pedestrian (V2P) communications^8^. The goal of beam management is to select the most appropriate beam pattern that can optimize communication performance based on the transmission environment. However, beam management becomes significantly more challenging due to the unique characteristics of V2V communication, such as high mobility.
3. Partial observation: Partial observation is a key characteristic that distinguishes vehicle-to-vehicle (V2V) communication from vehicle-to-infrastructure (V2I) communication. Unlike V2I, the positions of roadside infrastructures (RSIs) in V2V communication are stationary, and they regularly gather information from all nearby vehicles, resulting in more comprehensive observation information than other vehicles can provide. In optimization and control problems, such as beam management, global observation information plays a vital role. Vehicles, on the other hand, can only access a partial set of environmental information (such as channel information, the positions of other vehicles, and the location of RSIs) through their own sensors^9^. If observation information is insufficient, the vehicle may not accurately manage the beam pattern, leading to a decline in performance.

In V2V (vehicle-to-vehicle) scenarios, it is necessary to employ a beam management approach that can achieve low latency, high reliability, and high spectral efficiency communications, even in situations where the channel state is unstable, the transmitting and receiving nodes are in motion at high velocity, and only partial observation information is available. The 5G standard mandates that the transmitter/receiver selects a suitable beam for beam alignment and performs beam training on the selected beam to enhance its directionality, thus achieving a stronger beamforming gain during the initial connection setup between nodes^10^. Assuming four beam patterns are available for both the transmitter and receiver to select during the beam alignment phase, 16 (4×4) pairing operations are required to identify the optimal beam pattern. Subsequently, the transmitter/receiver needs to refine the selected beam using channel state information (CSI) obtained through a complex channel estimation method. However, the complexity of the 5G standard-based beam management approach renders it impractical for achieving low-latency V2V communications. The deep reinforcement learning (DRL) method presents a potential solution for V2V beam management.

By formulating the beam management problem as a Markov decision process, a reinforcement learning (RL) agent can be used to select the optimal beam pattern directly, without the need for an exhaustive search. Additionally, extending the conventional DRL approach to the multi-agent case allows for the sharing of observation information between vehicles, and even joint training, thereby improving the training efficiency and overall algorithm performance. In this paper, we propose a DRL-based beam management method for V2V communications. We first generated traffic flow data for a typical highway scenario using Anylogic simulation software, which was then used to train the DRL agent. Subsequently, we analyzed the statistical characteristics of the traffic flow data and identified self-similarity in the time domain. Finally, we adapted the DRL framework to leverage the self-similarity of the traffic flow data, resulting in improved algorithm performance. We also compared various DRL frameworks and found that the independent proximal policy optimization (IPPO) method is more effective for beam management in V2V scenarios. We provide a detailed discussion of this finding in the paper.

## Related work

In recent years, as 5G systems have been extensively adopted for commercial use, research on beam management for 5G NR FR2 frequency bands has gained significant attention from academia and industry. For instance, Ref.^10^ proposes a beam management algorithm that uses spatially distributed antenna subarrays, instead of a single co-located antenna array, to reduce beam alignment errors. This is achieved by minimizing the sum of squared errors between the estimated beam direction after the beam training process and the refined beam direction obtained from measured position and velocity data. In Ref.^11^, the authors propose a tractable mmWave communication model that considers both the distance and heading of vehicles, enabling low-complexity beam design. To optimize relay selection and beam management with minimal overhead, D.-Kim et al. formulate a sequential decision problem in Ref.^12^. Moreover, a machine learning (ML) approach is introduced in Ref.^13^ to achieve fast analog beam selection for mmWave V2V communications, thereby achieving higher data rates with significantly lower computational complexity. Ashraf et al. focus on feedback-based autonomous reconfiguration of the hypersurface controller states to establish a reliable communication channel between a transmitter and a receiver using programmable reflection on the hypersurface, specifically when there is no Line-of-sight (LoS) path between them^14^.

On the other hand, in order to overcome the limitations of regular beam management methods in terms of latency and reliability, some researchers have attempted to apply artificial intelligence methods in the field of beam management, and have achieved a series of significant results^15^. In Ref.^16^, a learning-based cost-efficient resource allocation algorithm using deep neural networks is proposed to ensure system performance while achieving cost-efficiency. Ref.^17^ proposes a deep reinforcement learning (DRL) based method to select unblocked UAV relays and perform beam management jointly. Hu et al. introduce a system for radio resource allocation in V2V communications that rely on the proximal strategy optimization method^18^. Tang et al. explores the channel model in high mobility and heterogeneous networks and puts forth a novel approach for radio resource allocation^19^. Specifically, a deep reinforcement learning-based intelligent time-division duplexing (TDD) configuration algorithm is proposed to allocate radio resources dynamically.

Although there has been a considerable amount of research focused on beam management in V2V scenarios in recent years, these studies often remain confined within the communication domain, neglecting the impact of vehicles’ mobility on beam management or, in cases where such an impact is acknowledged, relying on relatively simplistic mobility models to simulate vehicle movement. Furthermore, despite some efforts to employ AI techniques to address beam management in V2V scenarios, there has been insufficient research on the distribution of vehicle mobility or traffic data. Data distribution, as the foundation of AI data, determines the appropriate AI methods for problem-solving. Therefore, based on the above reasons, this paper makes the following contributions:

1. We presented a novel approach to addressing the beam management issue in V2V communication scenarios by utilizing a traffic flow dataset-based DRL framework. By carefully selecting the appropriate state, action, and reward structures, we significantly improved the algorithm’s effectiveness and enhanced the network’s overall performance in terms of spectral efficiency and reliability.
2. We analyzed the statistical characteristics of the traffic flow data and found that the data set has high self-similarity in the temporal dimension. Based on this observation, we introduced an RNN structure to the DRL framework to address this self-similarity, resulting in improved network performance in terms of spectral efficiency and reliability.
3. By analyzing the characteristics of the V2V communication scenario, we introduced the twin delayed deep deterministic policy gradient (TD3) model into the proposed DRL framework and found that the TD3 model is more suitable for V2V communication scenarios compared to the IPPO model. Combining the insights gained from points 2 and 3, we proposed the ITD3 with RNN framework. This framework optimizes beam management control to achieve spectral efficiency optimization in V2V scenarios while ensuring communication latency and reliability.

The rest of this paper is described as follows: “Network architecture” describes the network architecture of 5G NR FR2 based V2V communications. The beam management process and performance metrics are further described in “Performance evaluation”. “Deep reinforcement learning model” provides a detailed description of the DRL framework in this paper. The simulation results and the corresponding discussions are shown in “Results and discussions”, the potential future work is discussed in “Future work”, and conclusions are made in “Conclusion”.

## Network architecture

**Figure 1:**
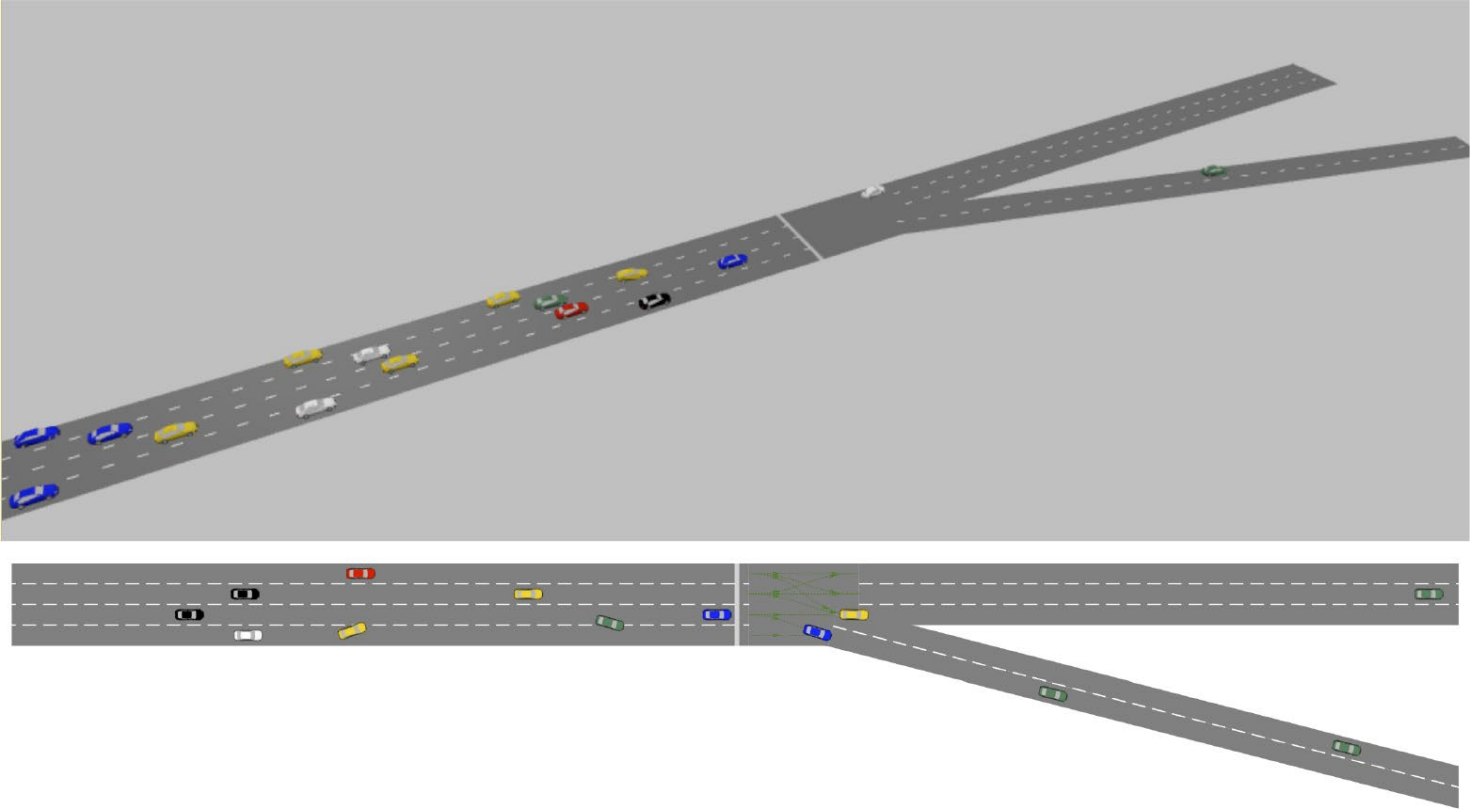
Network architecture (3D/2D).

In this study, we focus on a V2V network that utilizes agent-based simulation software, Anylogic 8.8, to simulate the mobility patterns (i.e., velocity and position) of vehicles (as shown in Fig. 1). Specifically, we select a typical highway scenario as the simulation area, which comprises four lanes and a two-lane exit, and define it as $\mathbb {A}$. To support beam management in the V2V network, we employ DRL technology, whereby a DRL agent determines the beam pattern of each vehicle at the beginning of each frame.

Following the 5G standard, the network in our study is configured to operate in a time-slotted manner with a time slot duration of 1ms and a frame length of 10 ms (i.e., consisting of 10 time slots). We define the set of vehicles within the simulation area $\mathbb {A}$ at time slot $t_i$ ($i \in \left[ {1,10} \right]$), frame $f_j$ ($j \in \left[ {1,\infty } \right)$) as ${\Phi _{i,j}} = \left\{ {{Vu_k}\left| {k = 1,2, \ldots ,N_{i,j}^{\textrm{V}}} \right. } \right\}$, where $N_{i,j}^{\textrm{V}}$ is the number of vehicles at time slot $t_i$, frame $f_j$. Without loss of generality, we chose a cluster consisting of two vehicles as a typical cluster, and the corresponding beam management method can be extended to clusters with more vehicles. For a given time slot $t_i$, frame $f_j$, define the typical cluster formed by vehicle $Vu_m$ and $Vu_n$ ($m \ne n$) as $G_{i,j}^{m,n}$.

**Figure 2:**
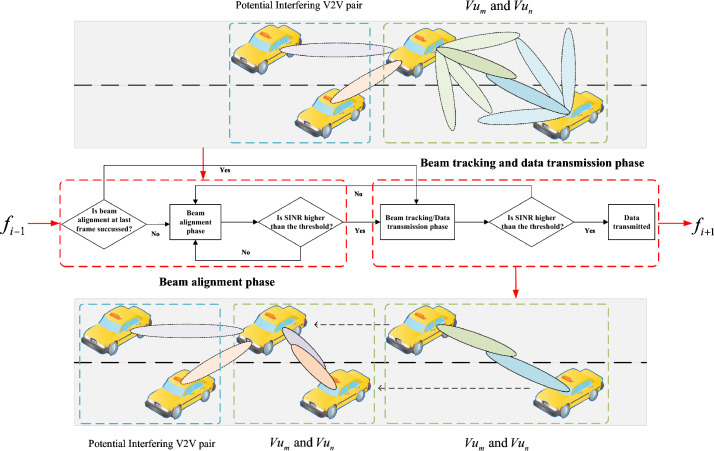
Communication process.

At the beginning of each frame (i.e., $t_0$ of $f_j$), vehicles contained in $G_{i,j}^{m,n}$ need to determine proper beam patterns to keep connected with each other. Defining the determined beam patterns of $Vu_m$ and $Vu_n$ at frame $f_j$ as ${\textbf{F}}_{j}^{m,n}$ and ${\textbf{F}}_{j}^{n,m}$, respectively. The beam alignment, beam tracking, and communication process is shown in Fig. 2. Recall that the network operates in a time-slotted manner. Also, we assume $Vu_m$ and $Vu_n$ need to change safety-critical information during each frame to maintain road safety. As shown in Fig. 2, the V2V communication process can be summarized into two phases;

1. Beam alignment phase: In this phase, vehicles contained in $G_{i,j}^{m,n}$ will use the 5G NR FR2 band to do beam alignment to make initials connection with each other. If beam alignment succeeds, i.e., $Vu_m$ and $Vu_n$ successfully capture each other with $B_{i,j}^m$ and $B_{i,j}^n$, the communication process will turn to the following beam tracking phase. Once $Vu_m$ or $Vu_n$ fails to track each other in the following beam tracking phase, the communication between $Vu_m$ and $Vu_n$ will be turned to the beam alignment phase. Here we defined the beam alignment phase as ${\mathscr {P}}_{\textrm{BA}}$.
2. Beam tracking and data transmission phase: In this phase, $Vu_m$ and $Vu_n$ will keep adjusting the beam direction and width to maintain the link quality between them. At each frame, the transmitter will first determine a proper beam pattern based on the previous feedback information from the receiver through uplink transmission every frame. Then, if the link quality is high enough, e.g., the channel capacity is higher than a given threshold $\gamma _{\textrm{th}}$, $Vu_m$ and $Vu_n$ will maintain the beam tracking phase and keep transmitting data. However, if the link quality drops (mostly because of an unpredicted large movement of $Vu_m$ and $Vu_n$), $Vu_m$ and $Vu_n$ will start a new beam alignment phase to re-capture each other. Similarly, we defined the beam tracking and data transmission phase as ${\mathscr {P}}_{\textrm{BT}}$

Hence, in a given frame $f_j$, the communication between $Vu_m$ and $Vu_n$ could be in the beam alignment phase or the beam tracking and data transmission phase, depending on their initial connection success and subsequent beam tracking process. As data transmission does not occur during the beam alignment phase, $Vu_m$ and $Vu_n$ need to avoid this phase to improve the long-term average spectral efficiency. However, their movement is unpredictable as they only have access to feedback information, such as location and velocity, from the previous time slots. Based on Ref.^20^, we assume that the location information from $Vu_m$ and $Vu_n$ has small normally distributed errors, denoted as ${e_{\textrm{lo}}}$ (${e_{\textrm{lo}}} \sim N(0,,{\sigma _{\textrm{lo}}})$). Even errors as small as several centimeters can significantly affect link performance in 5G scenario with massive MIMO technology, which requires a small beam width to compensate for high channel fading. Thus, repeating the beam alignment phase becomes necessary. To account for this, we model uplink transmission/decoding failures as a stationary stochastic process denoted by ${\Theta _{\textrm{F}}}$. ${\mathscr {N}}j$ represents the probability of such a failure occurring at $f_j$. Additionally, blockage by other vehicles could be significant at mmWave frequencies^21,22^. We model the blockage as a stationary stochastic process denoted by ${\Theta {\textrm{B}}}$, with ${\mathscr {M}}_j$ representing the probability of a blockage occurring at $f_j$. To address these issues, we propose a DRL-assisted beam tracking method, which is described in the following section.

## Performance evaluation

### Codebook-based beamforming

As we mentioned in the last section, in the beam alignment phase, $Vu_m$ and $Vu_n$ will set up an initial link through beam alignment. However, the link is not stable since $Vu_m$ and $Vu_n$ are probably moving with a high velocity. Thus, They need to keep adjusting the beam pattern to maintain an acceptable link quality. In this paper, the link quality is measured by the channel capacity, which is contained in the feedback message sent through the uplink. We assume that all vehicles are equipped with a uniform planar array (UPA) of ${M_x} \times {M_y}$ antenna elements. Since the duration of a time slot is as short as 1ms, the channel condition during each time slot is supposed to be stable, i.e., the channel matrix between $Vu_m$ and $Vu_n$ will not change during each time slot. Let’s take the situation where $Vu_m$ is the transmitter and $Vu_n$ is the receiver as an example for the following analysis. The derivation process for other situations is similar.

The channel between the transmit array and receiver array at time slot $t_i$ can be expressed by (1),

1 $$\begin{aligned} \begin{array}{l} {\textbf{H}}_{i,j}^{m,n} = \alpha _{{\textrm{LoS}}}^{m,n}\left( {{f_{\textrm{c}}},d_i^{m,n}} \right) {G_{{\textrm{TR}}}}{G_{{\textrm{AR}}}} {\varvec{\alpha }}_{{\textrm{AR}}}^{m,n}\left( {\theta _{{\textrm{AR}}}^{m,n}\left( i \right) ,\varphi _{{\textrm{AR}}}^{m,n}\left( i \right) } \right) {\varvec{\alpha }}_{{\textrm{TR}}}^{m,n}{\left( {\theta _{{\textrm{DP}}}^{m,n}\left( i \right) ,\varphi _{{\textrm{DP}}}^{m,n} \left( i \right) } \right) ^H}\quad \quad \;\;\;\\ \quad \quad + \sum \limits _{k = 1}^{{N_{{\textrm{pa}}}}} {\alpha _{{\textrm{NL}}}^{k,m,n}\left( {{f_{\textrm{c}}},d_i^{k,m,n}} \right) {G_{{\textrm{TR}}}}{G_{{\textrm{AR}}}} \cdot }{\varvec{\alpha }}_{{\textrm{AR}}}^{k,m,n}\left( {\theta _{{\textrm{AR}}}^{k,m,n}\left( i \right) ,\varphi _{{\textrm{AR}}}^{k,m,n}\left( i \right) } \right) {\varvec{\alpha }}_{{\textrm{TR}}}^{k,m,n}{\left( {\theta _{{\textrm{DP}}}^{k,m,n}\left( i \right) ,\varphi _{{\textrm{DP}}}^{k,m,n}\left( i \right) } \right) ^H}. \end{array} \end{aligned}$$

where ${G_{{\textrm{TR}}}}$ and ${G_{{\textrm{AR}}}}$ are the transmit and receive antenna gains; ${\varvec{\alpha }}_{{\textrm{TR}}}^{m,n}\left( \cdot \right)$ and ${\varvec{\alpha }}_{{\textrm{AR}}}^{m,n}\left( \cdot \right)$ represent the array steering vectors of $R{s_m}$ and $V{u_n}$, respectively; $\alpha _{{\textrm{LoS}}}^{m,n}\left( {{f_{\textrm{c}}},d_i^{m,n}} \right)$ and $\alpha _{{\textrm{NL}}}^{k,m,n}\left( {{f_{\textrm{c}}},d_i^{k,m,n}} \right)$ are the path losses of the LoS path and NLoS path, respectively; ${f_{\textrm{c}}}$ is the carrier frequency; $d_i^{m,n}$ is the length of the LoS path at time slot $t_i$; $d_i^{k,m,n}$ is the length of the *k*th NLoS path at time slot $t_i$; ${N_{{\textrm{pa}}}}$ represents the number of NLoS paths; $\theta _{{\textrm{DP}}}^{m,n}\left( i \right)$ and $\theta _{{\textrm{AR}}}^{m,n}\left( i \right)$ are the azimuth AoD and AoA of the LoS path at time slot $t_i$, respectively; $\theta _{{\textrm{AR}}}^{k,m,n}\left( i \right)$ and $\theta _{{\textrm{DP}}}^{k,m,n}\left( i \right)$ are the azimuth AoD and AoA of the *k*th NLoS path at time slot $t_i$, respectively. If a blockage occurs, the LoS path will be blocked, and only NLoS paths exist.

Similarly, $\varphi _{{\textrm{DP}}}^{m,n}\left( i \right)$/$\varphi _{{\textrm{AR}}}^{m,n}\left( i \right)$ is the elevation AoD/AoA of the LoS path at time slot $t_i$, and $\varphi _{{\textrm{AR}}}^{k,m,n}\left( i \right)$/$\varphi _{{\textrm{DP}}}^{k,m,n}\left( i \right)$ is the elevation AoD/AoA of the the *k*th NLoS path at time slot $t_i$. More specifically, the number of multipath components, $N_{\textrm{pa}}$, is a uniformly distributed variable within a range of [1,5]. For the azimuth AoD and AoA of the *k*th NLoS path at time slot $t_i$, i.e., $\theta _{{\textrm{DP}}}^{k,m,n}\left( i \right)$ and $\theta _{{\textrm{AR}}}^{k,m,n}\left( i \right)$, we have

2 $$\begin{aligned} \left\{ \begin{array}{l} \theta _{{\textrm{DP}}}^{k,m,n}\left( i \right) = \theta _{{\textrm{DP}}}^{m,n}\left( i \right) + \vartheta _{{\textrm{DP}}}^{k,m,n}\left( i \right) \\ \theta _{{\textrm{AR}}}^{k,m,n}\left( i \right) = \theta _{{\textrm{AR}}}^{m,n}\left( i \right) + \vartheta _{{\textrm{AR}}}^{k,m,n}\left( i \right) \end{array} \right. \end{aligned}$$

where $\vartheta _{{\textrm{DP}}}^{k,m,n}\left( i \right)$ and $\vartheta _{{\textrm{AR}}}^{k,m,n}\left( i \right)$ follow two independent uniform distributions on $\left[ { - \pi ,\;\pi } \right]$ (i.e., $\left[ {{\mathrm{- }}{{180}^ \circ },{{180}^ \circ }} \right]$ ). Similarly, for the elevation AoD and AoA of the *k*th NLoS path at time slot $t_i$, i.e., $\varphi _{{\textrm{DP}}}^{k,m,n}\left( i \right)$ and $\varphi _{{\textrm{AR}}}^{k,m,n}\left( i \right)$, we have

3 $$\begin{aligned} \left\{ \begin{array}{l} \varphi _{{\textrm{DP}}}^{k,m,n}\left( i \right) = \varphi _{{\textrm{DP}}}^{m,n}\left( i \right) + \psi _{{\textrm{DP}}}^{k,m,n}\left( i \right) \\ \varphi _{{\textrm{AR}}}^{k,m,n}\left( i \right) = \varphi _{{\textrm{AR}}}^{m,n}\left( i \right) + \psi _{{\textrm{AR}}}^{k,m,n}\left( i \right) \end{array} \right. \end{aligned}$$

where $\psi _{{\textrm{DP}}}^{k,m,n}\left( i \right)$ and $\psi _{{\textrm{AR}}}^{k,m,n}\left( i \right)$ follow two independent uniform distributions on $\left[ { - \pi /4,\;\pi /4} \right]$ (i.e., $\left[ {{\mathrm{- }}{{45}^ \circ },{{45}^ \circ }} \right]$). For ${M_x} \times {M_y}$-elements UPA, the array steering vector can be expressed by

4 $$\begin{aligned} \begin{array}{l} {{\textbf{a}}_{{\mathrm{{UPA}}}}}\left( {\theta ,\varphi } \right) = \left[ {1, \ldots ,{e^{J\pi \left( {{m_x}\sin \varphi \sin \theta + {m_y}\cos \theta } \right) }},} \right. {\left. { \ldots ,{e^{J\pi \left( {\left( {{M_x} - 1} \right) \sin \varphi \sin \theta + \left( {{M_y} - 1} \right) \cos \theta } \right) }}} \right] ^T}, \end{array} \end{aligned}$$

where $m_x$ and $m_y$ are the antenna elements index with $0 \le m_x \le M_x$ and $0 \le m_y \le M_y$, respectively; ${r_{\textrm{A}}} = {\lambda _{\textrm{C}}}/2$ is the antenna element spacing, *J* is the imaginary unit, ${\lambda _{\textrm{C}}}$ is the wavelength; $\theta$ and $\varphi$ are variables of the function ${{\textbf{a}}_{{\mathrm{{UPA}}}}}\left( {\theta ,\varphi } \right)$. Since the duration of a time slot is short, here we assume that the beam pattern, i.e., precoding vector, is reselected in every subframe instead of every time slot. Denoting $N_{\textrm{TR}}=M_x \times M_y$, $N_{\textrm{RC}}=N_x \times N_y$, based on Shannon equation, the normalized channel capacity (i.e., maximum achievable spectral efficiency) of the link at time slot $t_i$, frame $f_j$ can be expressed by^23^,

5 $$\begin{aligned} C_{i,j}^{m,n} = {\log _2}\det \left( {{{\textbf{I}}_{{N_{{\textrm{RC}}}}}} + \frac{{{P_{{\textrm{TR}}}}}}{{\sigma _{\textrm{s}}^{\textrm{2}}}}{\textbf{H}}_{i,j}^{m,n}{\textbf{F}}_j^{m,n}{{\left( {\overline{\textbf{J}} _{i,j}^{m,n}} \right) }^{ - 1}}{{\left( {{\textbf{F}}_j^{m,n}} \right) }^H}{{\left( {{\textbf{H}}_{i,j}^{m,n}} \right) }^H}} \right) , \end{aligned}$$

with

6 $$\begin{aligned} \overline{\textbf{J}} _{i,j}^{m,n} = {{\textbf{I}}_{{N_{{\textrm{RC}}}}}} + \sum \limits _{p = 1,p \ne m,n}^{N_{i,j}^{\textrm{V}}} {{\textbf{H}}_{i,j}^{p,q}{\textbf{F}}_j^{p,q}{{\left( {{\textbf{F}}_j^{p,q}} \right) }^H}{{\left( {{\textbf{H}}_{i,j}^{p,q}} \right) }^H}}. \end{aligned}$$

where $\overline{\textbf{J}} _{i,j}^{m,n}$ is the received interference matrix of V2V pair $V{u_m}$ and $V{u_n}$ at time slot ${t_i}$; frame ${f_j}$, ${\textbf{H}}_{i,j}^{p,q}$ is the channel matrix of the interfering V2V pair, e.g., $V{u_p}$ and $V{u_q}$, and ${\textbf{F}}_j^{p,q}$ is the corresponding precoding vector; $\sigma _{\textrm{s}}^{\textrm{2}}$ is the power of additive white Gaussian noise (AWGN) of the channel between the transmitter and receiver; ${{\textbf{I}}_{{N_{{\textrm{RC}}}}}}$ is an ${N_{{\textrm{RC}}}} \times {N_{{\textrm{RC}}}}$ identity matrix; $\det \left( \cdot \right)$ is the determinant of the given matrix; ${\textbf{F}}_{j}^{m,n}$ is the precoding vector for frame $f_j$ and ${P_{{\textrm{TR}}}}$ is the transmit power of the UPA. We use ${\textbf{F}}_{j}^{m,n}$ instead of ${\textbf{F}}_{i,j}^{m,n}$ here since the beam pattern is determined at the first time slot of $f_j$ and then remain stable before next frame, i.e., $f_{j+1}$.

**Figure 3:**
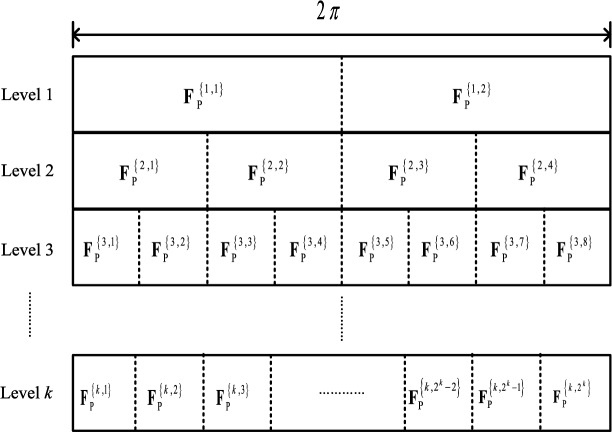
Structure of the codebook $\textbf{CB}$.

A common approach to change the beam pattern of $Vu_m$ is to set a threshold for the signal to interference plus noise ratio (SINR) of the current link. If the SINR is below the threshold, $Vu_m$ will switch to another beam pattern to improve the link quality; otherwise, it will maintain the existing one. Nevertheless, this technique is not suitable for V2V communication as high vehicle velocity can result in significant changes in link quality. To address this, an AI agent is employed to select ${\textbf{F}}_j^{m,n}$ from a pre-defined codebook, which controls the actions of $Vu_m$. The pre-defined codebook is depicted in Fig. 3.

The construction of the codebook can be found in Ref.^24^. Since the movement of $Vu_m$/$Vu_n$ is hard to predict, a codebook with the same beam width is not robust enough to handle all the situations that may occur during beam tracking. This is why we have used a multi-level codebook. For example, a codeword ${\textbf{F}}_{\textrm{P}}^{\left\{ {a,b} \right\} }$ in the codebook will be selected by the RL agent to do beam tracking and data transmission in each frame $f_j$, i.e., ${\textbf{F}}_{\textrm{P}}^{\left\{ {a,b} \right\} } \rightarrow {\textbf{F}}_j^{m,n}$ in (5). The parameter *a* denotes the level of the codeword and *b* is the location of the codeword at level *a*. Since the vehicles’ dynamics on elevation is relatively small compared with the azimuth cases, we do not consider the variation of elevation AoD and AoA in this paper. Then, the corresponding beam width and beam direction of ${\textbf{F}}_{\textrm{P}}^{\left\{ {a,b} \right\} }$ can be expressed by

7 $$\begin{aligned} \left\{ \begin{array}{l} B_{\textrm{W}}^{a,b} = \frac{\pi }{{{2^a}}}\\ B_{\textrm{D}}^{a,b} = \frac{\pi }{2} - \frac{{b\pi }}{{{2^a}}} \end{array} \right. \quad . \end{aligned}$$

To improve the beamforming gain and compensate for excessive path losses at higher frequencies, a codeword in the lower level of the codebook is not considered for beam tracking and data transmission. Thus, in this paper, vehicles only use the codeword from levels 3 to 6 of the codebook to do beam tracking and data transmission. Based on (7), the corresponding beam width is from $2\pi /8 = {22.5^ \circ }$ to $2\pi /64 = {1.40625^ \circ }$^25^.

### Problem formulating

Based on (5), we define the SINR of the transmitting link from $Vu_m$ to $Vu_n$ at time slot $t_i$, frame $f_j$ as ${\rho _{i,j}^{m,n}}$. Similarly, the SINR of the transmitting link from $Vu_n$ to $Vu_m$ is defined by ${\rho _{i,j}^{n,m}}$. By defining the communication phase of $Vu_m$ at $t_i$, $f_j$ as ${{\mathscr {R}}_{i,j}^{m,n}}$, the long-term overall achievable spectral efficiency can be expressed by

8 $$\begin{aligned} {{\overline{C}} _{{\textrm{ov}}}} = \sum \limits _{j = 1}^\infty {\sum \limits _{i = 1}^{10} {\left( {C_{i,j}^{m,n}{\textbf{1}}\left( {{\mathscr {R}}_j^{m,n} \in {{\mathscr {P}}_{{\textrm{BT}}}}} \right) {\textbf{1}}\left( {\rho _{i,j}^{m,n} \ge {\gamma _{{\textrm{th}}}}} \right) + C_{i,j}^{n,m}{\textbf{1}}\left( {{\mathscr {R}}_j^{n,m} \in {{\mathscr {P}}_{{\textrm{BT}}}}} \right) {\textbf{1}}\left( {\rho _{i,j}^{n,m} \ge {\gamma _{{\textrm{th}}}}} \right) } \right) } } \quad, \end{aligned}$$

where ${\textbf{1}}\left( \cdot \right)$ is the indicator function, $\gamma _{{\textrm{th}}}$ is the SINR threshold for the received signal to be successfully decoded. Based on (8), we can see that the value of ${\overline{C}} _{{\textrm{ov}}}$ is related to the value of $C_{i,j}^{m,n}$/$C_{i,j}^{n,m}$, $\rho _{i,j}^{m,n}$/$\rho _{i,j}^{n,m}$, and total number of ${\mathscr {P}}_{{\textrm{BT}}}$. Furthermore, based on (5), the value of $C_{i}^{m,n}$/$C_{i}^{n,m}$ depends on the channel condition ${\textbf{H}}_{i,j}^{m,n}$/${\textbf{H}}_{i,j}^{m,n}$ and the corresponding beam pattern determined by $Vu_m$/$Vu_n$, i.e., ${\textbf{F}}_{j}^{m,n}$/${\textbf{F}}_{j}^{n,m}$. The total number of ${\mathscr {P}}_{{\textrm{BT}}}$ is also related to the beam pattern selected by $Vu_m$/$Vu_n$ during the beam alignment/beam tracking phase. Therefore, optimizing the overall spectral efficiency actually means choosing the appropriate beam pattern for the channel conditions. However, it is difficult to obtain channel conditions in the scenario where the vehicles are moving at high velocity and using massive MIMO for communication. Thus, we model the beam management problem in V2V communication as a Markov decision process (MDP) and then use a DRL-based method to solve it. The corresponding problem can be formulated by

9 $$\begin{aligned} { \begin{array}{*{20}{l}} {{\textrm{maximize}}\quad \quad {{\bar{C}}_{{\textrm{ov}}}}\left( k \right) = \sum \limits _{j = 1}^k {\sum \limits _{i = 1}^{10} {\left( {C_{i,j}^{m,n}{\textbf{1}}\left( {\left( {R_j^{m,n} \in {P_{{\textrm{BT}}}}} \right) \wedge \left( {\rho _{i,j}^{m,n} \ge {\gamma _{{\textrm{th}}}}} \right) } \right) + C_{i,j}^{n,m}{\textbf{1}}\left( {\left( {R_j^{n,m} \in {P_{{\textrm{BT}}}}} \right) \wedge \left( {\rho _{i,j}^{n,m} \ge {\gamma _{{\textrm{th}}}}} \right) } \right) } \right) } }.}\\ {{\textrm{subject}}{\hspace{0.55542pt}} {\textrm{to}}\quad \quad C_{i,j}^{m,n} = {\log _2}\det \left( {{{\textbf{I}}_{{N_{{\textrm{RC}}}}}} + \frac{{{P_{{\textrm{TR}}}}}}{{\sigma _{\textrm{s}}^{\textrm{2}}}}{\textbf{H}}_{i,j}^{m,n}{\textbf{F}}_j^{m,n}{{\left( {\overline{\textbf{J}} _{i,j}^{m,n}} \right) }^{ - 1}}{{\left( {{\textbf{F}}_j^{m,n}} \right) }^H}{{\left( {{\textbf{H}}_{i,j}^{m,n}} \right) }^H}} \right) }\\ {\quad \quad \quad \quad \quad \;\;\;C_{i,j}^{n,m} = {\log _2}\det \left( {{{\textbf{I}}_{{N_{{\textrm{RC}}}}}} + \frac{{{P_{{\textrm{TR}}}}}}{{\sigma _{\textrm{s}}^{\textrm{2}}}}{\textbf{H}}_{i,j}^{n,m}{\textbf{F}}_j^{n,m}{{\left( {\overline{\textbf{J}} _{i,j}^{n,m}} \right) }^{ - 1}}{{\left( {{\textbf{F}}_j^{n,m}} \right) }^H}{{\left( {{\textbf{H}}_{i,j}^{n,m}} \right) }^H}} \right) \quad \quad }\\ {\quad \quad \quad \quad \quad \;\;\;\rho _{i,j}^{m,n} = \frac{{{P_{{\textrm{TR}}}}}}{{\sigma _{\textrm{s}}^{\textrm{2}}}}{\textbf{H}}_{i,j}^{m,n}{\textbf{F}}_j^{m,n}{{\left( {\overline{\textbf{J}} _{i,j}^{m,n}} \right) }^{ - 1}}{{\left( {{\textbf{F}}_j^{m,n}} \right) }^H}{{\left( {{\textbf{H}}_{i,j}^{m,n}} \right) }^H}}\\ {\quad \quad \quad \quad \quad \;\;\;\rho _{i,j}^{n,m} = \frac{{{P_{{\textrm{TR}}}}}}{{\sigma _{\textrm{s}}^{\textrm{2}}}}{\textbf{H}}_{i,j}^{n,m}{\textbf{F}}_j^{n,m}{{\left( {\overline{\textbf{J}} _{i,j}^{n,m}} \right) }^{ - 1}}{{\left( {{\textbf{F}}_j^{n,m}} \right) }^H}{{\left( {{\textbf{H}}_{i,j}^{n,m}} \right) }^H}}\\ {\quad \quad \quad \quad \quad \;\;\;\overline{\textbf{J}} _{i,j}^{m,n} = {{\textbf{I}}_{{N_{{\textrm{RC}}}}}} + \sum \limits _{p = 1,p \ne m,n}^{N_{i,j}^{\textrm{V}}} {{\textbf{H}}_{i,j}^{p,q}{\textbf{F}}_j^{p,q}{{\left( {{\textbf{F}}_j^{p,q}} \right) }^H}{{\left( {{\textbf{H}}_{i,j}^{p,q}} \right) }^H}}}\\ {\quad \quad \quad \quad \quad \;\;\;\overline{\textbf{J}} _{i,j}^{n,m} = {{\textbf{I}}_{{N_{{\textrm{RC}}}}}} + \sum \limits _{p = 1,p \ne n,m}^{N_{i,j}^{\textrm{V}}} {{\textbf{H}}_{i,j}^{p,q}{\textbf{F}}_j^{p,q}{{\left( {{\textbf{F}}_j^{p,q}} \right) }^H}{{\left( {{\textbf{H}}_{i,j}^{p,q}} \right) }^H}}}\\ {\quad \quad \quad \quad \quad \;\;\;{{\textbf{F}}_j^{m,n},\;{\textbf{F}}_j^{n,m} \in {\textbf{CB}}}.} \end{array} } \end{aligned}$$

where $\textbf{CB}$ is the codebook used for hybrid precoding, and $\wedge$ is the “and” operation.

## Deep reinforcement learning model

### Basic framework

We choose independent proximal policy optimization (IPPO)^26^ as the basic DRL framework to solve (9). Different from the regular single-agent PPO or multi-agent PPO (MAPPO), IPPO is a multi-agent reinforcement learning algorithm that modifies the PPO algorithm to handle environments with multiple agents without sharing parameters and policies among the agents. The IPPO uses a centralized value function to help agents learn more effective policies by allowing them to reason about the behavior of other agents. It also uses a decentralized policy optimization strategy that allows agents to update their policies independently. We chose IPPO instead of MAPPO based on the following reasons: 1) The V2V network is a typical distributed network lacking a central control mechanism for vehicle communication management; 2) The topology of V2V network changes rapidly, and the vehicles within a cluster are not fixed. Therefore, the MAPPO method is not suitable for such an environment; 3) The MAPPO used a specific mechanism of sharing parameters among agents, and this mechanism will increase the transmission delay, which is unacceptable in V2V communication.

Thus, we use an IPPO method to solve (9), in which each agent uses the same DRL framework with a shared reward function and independent parameters.

Let’s take $Vu_m$ as an example. The state of the environment at $t_i$, $f_j$ is defined by

10 $$\begin{aligned} {{\textbf{s}}_{i,j}} = \left[ {x_{i,j}^1,y_{i,j}^1,v_{i,j}^1,x_{i,j}^2,y_{i,j}^2,v_{i,j}^2, \cdots ,x_{i,j}^k,y_{i,j}^k,v_{i,j}^k, \cdots ,x_{i,j}^{N_{i,j}^{\textrm{V}}},y_{i,j}^{N_{i,j}^{\textrm{V}}},v_{i,j}^{N_{i,j}^{\textrm{V}}}} \right], \end{aligned}$$

where ${x_{i,j}^k}$, ${y_{i,j}^k}$, ${v_{i,j}^k}$ are the x-coordinate, y-coordinate, and velocity of $Vu_k$, respectively.

Based on (10), the local observation of $Vu_m$ at $t_i$, $f_j$ is defined by

11 $$\begin{aligned} {\textbf{o}}_{i,j}^{m,n}\left( {{{\textbf{s}}_{i,j}}} \right) = \left[ {{{\left( {x_{i,j}^m} \right) }^*},{{\left( {y_{i,j}^m} \right) }^*},v_{i,j}^m,{{\left( {x_{i,j}^n} \right) }^ \times },{{\left( {y_{i,j}^n} \right) }^ \times },{{\left( {v_{i,j}^n} \right) }^ \times }} \right]. \end{aligned}$$

Since $Vu_m$ achieves its own location information through a localization system (e.g., GPS), here ${{\left( {x_{i,j}^m} \right) }^*}$ and ${{\left( {y_{i,j}^m} \right) }^*}$ are the values of x-coordinate and y-coordinate provided by corresponding localization system. Based on Ref.^20^, we have

12 $$\begin{aligned} \left\{ \begin{array}{l} {\left( {x_{i,j}^m} \right) ^*} = x_{i,j}^m + {e_{{\textrm{lo}}}}\\ {\left( {y_{i,j}^m} \right) ^*} = y_{i,j}^m + {e_{{\textrm{lo}}}} \end{array} \right. . \end{aligned}$$

On the other hand, $Vu_m$ can obtain the mobility information of $Vu_n$ through sensing technology. Similarly, we assume that the information obtained by sensing has a normally distributed error ${e_{{\textrm{st}}}}$, where ${e_{\textrm{lo}}} \sim N(0,\,{\sigma _{\textrm{st}}})$. The accuracy of mobility information obtained through sensing is generally higher than that obtained through localization systems; for this reason, we assume ${\sigma _{\textrm{st}}}$ to be less than ${\sigma _{\textrm{lo}}}$. Thus, we have

13 $$\begin{aligned} \left\{ \begin{array}{l} {\left( {x_{i,j}^n} \right) ^ \times } = x_{i,j}^n + {e_{{\textrm{st}}}}\\ {\left( {y_{i,j}^n} \right) ^ \times } = y_{i,j}^n + {e_{{\textrm{st}}}}\\ {\left( {v_{i,j}^n} \right) ^ \times } = v_{i,j}^n + {e_{{\textrm{st}}}} \end{array} \right. . \end{aligned}$$

Since the observation obtained by $Vu_n$ is also shared in $G_{i,j}^{m,n}$. Based on (12)(13), the joint observation of $Vu_m$ can be expressed by

14 $$\begin{aligned} {\textbf{o}}_{i,j}^{m,n}\left( {{{\textbf{s}}_{i,j}}} \right) = \left[ {{{\left( {x_{i,j}^m} \right) }^*},{{\left( {y_{i,j}^m} \right) }^*},v_{i,j}^m,{{\left( {x_{i,j}^n} \right) }^ \times },{{\left( {y_{i,j}^n} \right) }^ \times },{{\left( {v_{i,j}^n} \right) }^ \times },{{\left( {x_{i,j}^n} \right) }^*},{{\left( {y_{i,j}^n} \right) }^*},v_{i,j}^n,{{\left( {x_{i,j}^m} \right) }^ \times },{{\left( {y_{i,j}^m} \right) }^ \times },{{\left( {v_{i,j}^m} \right) }^ \times }} \right] \quad. \end{aligned}$$

The action selected by $Vu_m$ for $Vu_n$ at time slot $t_i$, frame $f_j$ is defined by ${\textbf{a}}_{i,j}^{m,n}$. Since the number of codewords contained in $\textbf{CB}$ is fixed and discrete, we choose to use a discrete action space that ${\textbf{a}}_{i,j}^{m,n} \in \left[ {1,\sum \limits _{k = 3}^6 {{2^k}} } \right]$. For the sake of convenience, we map ${\textbf{F}}_{\textrm{P}}^{a,b}$ to ${\textbf{a}}_{i,j}^{m,n} \in \left[ {1,\sum \limits _{k = 3}^6 {{2^k}} } \right]$ as ${\textbf{F}}_{\textrm{P}}^{a,b} \rightarrow {\textbf{a}}_{i,j}^{m,n} = \left( {{2^a} - {2^3}} \right) + b$. Moreover, based on (8) and (9), the reward of time slot $t_i$ (frame $f_j$) is defined by

15 $$\begin{aligned} {r_{i,j}} = C_{i,j}^{m,n}{\textbf{1}}\left( {{\mathscr {R}}_j^{m,n} \in {{\mathscr {P}}_{{\textrm{BT}}}}} \right) {\textbf{1}}\left( {\rho _{i,j}^{m,n} \ge {\gamma _{{\textrm{th}}}}} \right) + C_{i,j}^{n,m}{\textbf{1}}\left( {{\mathscr {R}}_j^{n,m} \in {{\mathscr {P}}_{{\textrm{BT}}}}} \right) {\textbf{1}}\left( {\rho _{i,j}^{n,m} \ge {\gamma _{{\textrm{th}}}}} \right) . \end{aligned}$$

By defining the updating condition as ${\textbf{CD}}$, i.e., if ${\textbf{CD}}$ is satisfied, the NNs in the DRL framework will be updated. The pseudocode of the training process is shown in Algorithm 1.

**Algorithm 1:**
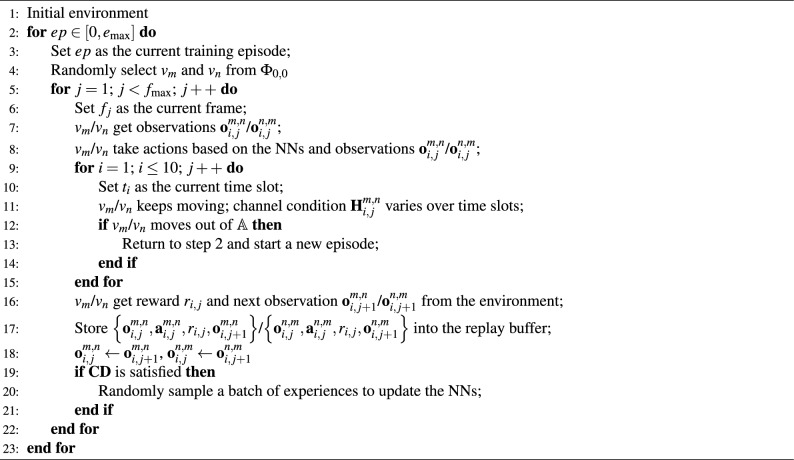
Training process of IPPO.

**Figure 4:**
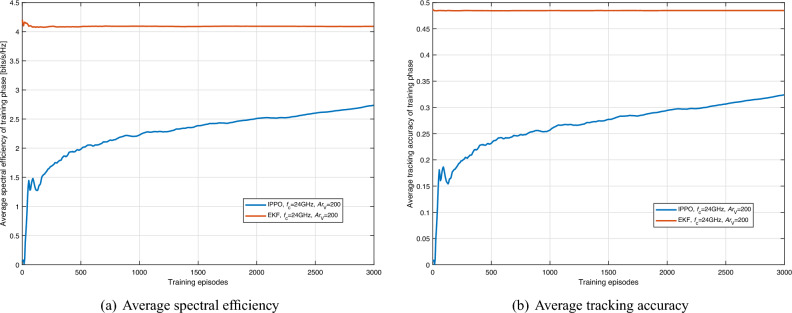
Average spectral efficiency and tracking accuracy of training phase (IPPO).

For the training phase, we generated 3000 episodes of training data, each containing 2000 training steps. Thus, the total data for training is $3000 \times 2000 = 6 \times {10^6}$. We employed the Anylogic 8.8 to create synthetic V2V communication scenarios, mimicking real-world vehicular movements, densities, and communication challenges. Furthermore, we generated another set of data for testing, and the total amount of the testing data is $100 \times 2000 = 2 \times {10^5}$. Representing the tracking accuracy as ${\rho _{i,j}^{m,n} \ge {\gamma _{{\textrm{th}}}}} \wedge {\rho _{i,j}^{n,m} \ge {\gamma _{{\textrm{th}}}}}$, the performance of the proposed framework is shown in Fig. 4. Here we choose the EKF-assisted method as the baseline, i.e., the beam management of $Vu_m$ and $Vu_n$ is determined based on the location information, which is predicted by a well-trained EKF. Let us take $Vu_m$ as an example. Specifically, the EKF used by $Vu_m$ will provide a prediction for the location of $Vu_n$ at the first time slot of $f_j$. Based on the prediction, $Vu_m$ will use the corresponding codeword located at the last level of $\textbf{CB}$ to do beam management.

The actor-network and critic-network used in the IPPO framework consist of three hidden layers, each having 300 neurons. The PPO clip parameter is set to 0.2. The activation functions used in the NNs are hyperbolic tangent function (tanh) functions. The capacity of the replay memory is 10000, and the batch size is 256. The learning rate is set as 0.0001, and the discounted factor is set at 0.99. The optimizer used in the IPPO framework is the Adam optimizer with $\epsilon =0.00001$. The maximum number of iterations in each episode (i.e., $f_{\textrm{max}}$) is 2000. As we can see from Fig. 4, the performance of the DRL framework keeps improving during the training phase and begin to converge after 3000 episodes. However, the performance is not acceptable according to the requirements of the 5G standard, particularly with regard to tracking accuracy, i.e., reliability. Therefore, it is necessary to make modifications to the basic framework to enhance its performance and bring it in line with the requirements of the 5G standard.

### State and reward

For DRL methods, the performance heavily relies on the definition of state and reward. Reasonably mapping environmental information and agent behaviors to state and reward can significantly increase the learning efficiency of the RL agent; however, if the state and reward are not properly defined, the policy of the agent may not even converge. Since the performance of the basic framework is not acceptable, we need to modify the structure of state and reward to improve the performance of V2V communication.

1. State: Keeping the variance of the state in a relatively small range can effectively improve the training results. At the same time, the relative positions of $Vu_m$ and $Vu_n$ are more important for beam management than their absolute positions. Therefore, we adjust (14) to (16),
  16 $$\begin{aligned} \begin{array}{l} {\textbf{o}}_{i,j}^{m,n}\left( {{{\textbf{s}}_{i,j}}} \right) = \left[ {{{\left( {x_{i,j}^m} \right) }^*} - {{\left( {x_{i,j}^n} \right) }^ \times },{{\left( {y_{i,j}^m} \right) }^*} - {{\left( {y_{i,j}^n} \right) }^ \times },v_{i,j}^m,{{\left( {x_{i,j}^n} \right) }^ \times } - {{\left( {x_{i,j}^m} \right) }^ \times },{{\left( {y_{i,j}^n} \right) }^ \times } - {{\left( {y_{i,j}^m} \right) }^ \times },{{\left( {v_{i,j}^n} \right) }^ \times }} \right. \\ \left. {\quad \quad \quad \quad \quad {{\left( {x_{i,j}^n} \right) }^*} - {{\left( {x_{i,j}^m} \right) }^ \times },{{\left( {y_{i,j}^n} \right) }^*} - {{\left( {y_{i,j}^m} \right) }^ \times },v_{i,j}^n,{{\left( {x_{i,j}^m} \right) }^ \times } - {{\left( {x_{i,j}^n} \right) }^ \times },{{\left( {y_{i,j}^m} \right) }^ \times } - {{\left( {y_{i,j}^n} \right) }^ \times },{{\left( {v_{i,j}^m} \right) }^ \times }} \right]. \end{array} \end{aligned}$$
  Do note that, based on the architecture of the network, the maximum and minimum value of $v_{i,j}^m$/$v_{i,j}^n$ is 20m/s and 30m/s, the maximum and minimum value of $\left| {x_{i,j}^m - x_{i,j}^n} \right|$ is 490 and 10 (ignore the additive error term), the maximum and minimum value of $\left| {y_{i,j}^m - y_{i,j}^n} \right|$ is 12 and 4 (ignore the additive error term).
2. Reward: According to the 5G standard, both the transmitter and the receiver must successfully complete beam alignment before the beam tracking and data transmission phase (see (15)). This means that the reward space is sparse, and a sparse reward space will reduce the efficiency of agent training. Therefore, we adjusted the definition of the reward so that it can better learn from failed experiences.
  17 $$\begin{aligned} \begin{array}{l} {r_{i,j}} = C_{i,j}^{m,n}{\textbf{1}}\left( {R_j^{m,n} \in {P_{{\textrm{BT}}}}} \right) {\textbf{1}}\left( {\rho _{i,j}^{m,n} \ge {\gamma _{{\textrm{th}}}}} \right) + C_{i,j}^{n,m}{\textbf{1}}\left( {R_j^{n,m} \in {P_{{\textrm{BT}}}}} \right) {\textbf{1}}\left( {\rho _{i,j}^{n,m} \ge {\gamma _{{\textrm{th}}}}} \right)  \\ - \quad \quad \frac{{{{{\bar{C}}}_{{\textrm{ov}}}}\left( {j - 1} \right) }}{{10\left( {j - 1} \right) }}\left( {{\textbf{1}}\left( {\left( {{\mathscr {R}}_j^{m,n} \notin {{\mathscr {P}}_{{\textrm{BT}}}}} \right) \vee \left( {\rho _{i,j}^{m,n} \le {\gamma _{{\textrm{th}}}}} \right) } \right) + {\textbf{1}}\left( {\left( {{\mathscr {R}}_j^{n,m} \notin {{\mathscr {P}}_{{\textrm{BT}}}}} \right) \vee \left( {\rho _{i,j}^{n,m} \le {\gamma _{{\textrm{th}}}}} \right) } \right) } \right). \end{array} \end{aligned}$$
  where ${{{{\bar{C}}}_{{\textrm{ov}}}}\left( {j - 1} \right) }$ is the average achievable spectral efficiency for the last $j-1$ frames.

### Dependency analysis

Another possible reason for the unacceptable performance is that the neural networks (NNs) used in the DRL model do not match the task. (1), The relationship between observation ${\textbf{o}}_{i,j}^{m,n}\left( {{{\textbf{s}}_{i,j}}} \right)$, action ${\textbf{a}}_{i,j}^{m,n}$, reward $r_{i,j}$, and next observation ${\textbf{o}}_{i,j+1}^{m,n}\left( {{{\textbf{s}}_{i,j+1}}} \right)$, is too complicated, which makes the PPO framework with a simple structure incapable of handling it; (2), the structure of the training data is too complex to handle for regular fully connected NNs. To improve the performance of the beam tracking accuracy and data transmission capacity, we analyze the structure of the training data. Since the action at each frame is selected based on the current observation ${\textbf{o}}_{i,j}^{m,n}\left( {{{\textbf{s}}_{i,j}}} \right)$ and previous experiences (also including previous observations), we choose data of observations to make the analysis. If the data has a strong temporal dependence, then recurrent neural networks (RNNs) may be better than fully connected NNs to solve the problem in (9).

The Hurst exponent is commonly utilized to analyze the dependence in a given dataset. It represents a measure of a time series’ long-term memory. Studies incorporating the Hurst exponent were originally developed in hydrology to address practical concerns related to determining the optimal size of dams for the unpredictable rain and drought conditions of the Nile River that had been observed over an extended period.^27^.

Based on^28^, the Hurst exponent can be estimated using three typical methods: (1) the Periodogram method; (2) the Variance-Time Analysis method; (3) and the Rescaled Adjusted Range Statistic (R/S) method. Here we choose the R/S method to evaluate the Hurst exponent of the training data. For a given series ${{\mathscr {X}}_i}$, we define the partial sum of ${{\mathscr {X}}_i}$ as,

18 $$\begin{aligned} {\mathscr {Y}}\left( k \right) = \sum \limits _{i = 1}^k {{{\mathscr {X}}_i}}, \end{aligned}$$

and sample variance is denoted by,

19 $$\begin{aligned} {{\mathscr {S}}^2}\left( k \right) = \frac{{\sum \limits _{i = 1}^k {{{\mathscr {X}}_i}} - \frac{{{\mathscr {Y}}{{\left( k \right) }^2}}}{k}}}{k},\quad k \ge 1. \end{aligned}$$

Furthermore, the R/S statistic is defined as,

20 $$\begin{aligned} \begin{array}{l} \frac{{{\mathscr {R}}\left( k \right) }}{{{\mathscr {S}}\left( k \right) }} = \frac{{\mathop {\max }\limits _{0 \le h \le k} \left( {0,{\mathscr {Y}}\left( h \right) - \frac{h}{k}{\mathscr {Y}}\left( k \right) } \right) }}{{{\mathscr {S}}\left( k \right) }} - \\ \quad \quad \quad \;\frac{{\mathop {\min }\limits _{0 \le h \le k} \left( {0,{\mathscr {Y}}\left( h \right) - \frac{h}{k}{\mathscr {Y}}\left( k \right) } \right) }}{{{\mathscr {S}}\left( k \right) }},\quad k \ge 1. \end{array} \end{aligned}$$

A log-log plot of the R/S statistic versus the number of points of the aggregated series should be a straight line with the slope being an estimate of the Hurst parameter. A value ${\mathscr {H}}$ in the range $\left( {0.5,\;1.0} \right)$ indicates that ${{\mathscr {X}}_i}$ has long-term positive self-correlation, meaning that more high values are expected in the series. A value in the range $\left( {0,\;0.5} \right)$ indicates that ${{\mathscr {X}}_i}$ has a long-term switching behavior between high and low values in adjacent pairs. This indicates that a single high value is likely to be followed by a low value. Also, this tendency to switch between high and low values will continue over a long period of time.

We utilized this method to evaluate the Hurst exponent of both position and velocity data of $V{u_n}$. The Hurst exponent for velocity and position data are 0.19 and 0.89, respectively. The Hurst exponent for velocity is close to 0 since it is more likely for a high-velocity vehicle to decrease its velocity, and vice versa. Conversely, the Hurst exponent for the data of position is close to 1 because the vehicle keeps moving in the same direction in the simulation scenario (and in most application scenarios). The Hurst exponent results indicate that the training data series has a long-term dependency, which corroborates the use of RNN models, such as LSTM/GRU, to improve the performance of the DRL framework.

### ITD3 framework with RNN

By analyzing the relations between reward and action, we found that the optimized reward is often obtained with an action close to the boundary. This point is similar to tasks in the field of robot control, where the TD3 model is commonly used to handle such a task. In the TD3 model, the target policy network and action policy network are separate, and their parameters are updated independently. The advantage of doing so is that overestimation can be reduced, but it may also cause the agent more prefer to choose edge actions. This may be disadvantageous in other tasks, but it can be used to solve V2V beam management problems. Since the locations of the vehicles are restricted on the lanes of the road, there is a high probability that $Vu_m$ and $Vu_n$ will be in the same lane. In such cases, the edge policy has a higher probability of achieving better performance. Here we choose to use the ITD3 framework with gate recurrent unit (GRU) to solve (9).

In regular reinforcement learning frameworks, randomly picking up ${N_{{\textrm{ba}}}}$ experiences from the replay buffer is a common way to construct a mini-batch. However, this approach does not work when using RNNs due to the temporal dependency requirement for its training data. Thus, we need to alter the standard procedure of constructing a mini-batch. The changes can include: (1) randomly selecting an experience $Exp_k$ from the replay buffer; (2) selecting the subsequent ${N_{{\textrm{ba}}}}/{N_{{\textrm{sba}}}} - 1$ experiences from the replay buffer to create a sub-batch, i.e., $\left\{ {Ex{p_k},Ex{p_{k + 1}}, \cdots ,Ex{p_{k + \frac{{{N_{{\textrm{ba}}}}}}{{{N_{{\textrm{sba}}}}}} - 1}},Ex{p_{k + \frac{{{N_{{\textrm{ba}}}}}}{{{N_{{\textrm{sba}}}}}}}}} \right\}$; (3) repeating steps (1) and (2) for ${N_{{\textrm{sba}}}}$ times to create a mismatch with ${N_{{\textrm{ba}}}}$ experiences. By following this procedure, the ITD3 framework can maximize the use of the GRU network and improve the performance of the agent for reinforcement learning. Since the TD3-based DRL framework requires a continuous action space, we map the level and direction of the codeword in $\mathbb{C}\mathbb{B}$ to the action as ${\textbf{a}}_{i,j}^{m,n} = \left\{ {a_{i,j}^{m,n},b_{i,j}^{m,n}} \right\}$, where $a_{i,j}^{m,n}$ and $b_{i,j}^{m,n}$ are equal to the *a* and *b* in ${\textbf{F}}_{\textrm{P}}^{{\mathrm{\{ }}a,b{\mathrm{\} }}}$, respectively.

In this paper, the normally distributed noise with a mean value of 0 and variance of 0.1 is added to the target action. In addition, no dropout and batch normalization is used. This is because both are not suitable for the DRL framework with RNN. Also, the policy is updated for every two Q-function updates.

**Table 1:** Definition and corresponding values of the parameter of simulations.

Symbol	Definition	Value
${L_{\textrm{R}}}$	Length of the road	500 m
${W_{{\textrm{la}}}}$	Width of the lane	4 m
${Ar_{\textrm{V}}}$	Arrival rate of vehicles	200 to 300/minute
${\sigma _{\textrm{v}}}$	Preferred velocity	20 to 30 m/s
${\sigma _{{\textrm{lo}}}}$	Variance of ${e_{\textrm{lo}}}$	0.1 m
${P_{{\textrm{TR}}}}$	Transmit power of vehicle	0.1 to 0.3 W
${N_{{\textrm{TR}}}}$	Number of transmit antennas	$64\times 16$
${N_{{\textrm{RC}}}}$	Number of receive antennas	$4\times 4$
${\gamma _{{\textrm{th}}}}$	SINR threshold	10 dB
${N_{{\textrm{ba}}}}$	Capacity of minibatch	256
${N_{{\textrm{sba}}}}$	Capacity of sub-minibatch	32
${\sigma _{{\textrm{ac}}}}$	Variance of the normally distributed exploration noise (ITD3)	0.5
${\sigma _{{\textrm{po}}}}$	Variance of the normally distributed policy noise (ITD3)	0.5
${l _{{\textrm{ac}}}}$	Learning rate of actor-network (ITD3)	0.0003
${l _{{\textrm{cr}}}}$	Learning rate of critic-network (ITD3)	0.0005
${\gamma _{\textrm{D}}}$	Discount factor (ITD3)	0.95

## Results and discussions

We carried out our simulations according to the parameters defined in Table 1. Based on the 5G standard, the maximum carrier bandwidth of FR2 is 200 MHz. Thus, during the training and testing process, the spectrum of the network is randomly chosen as 200$\cdot N_{i,j}^{\textrm{V}}$MHz bandwidth (i.e, from 24GHz to 71GHz: 200 MHz bandwidth per VU). Do note that the defaulted transmit power is 0.1W and may vary according to the simulation conditions.

**Figure 5:**
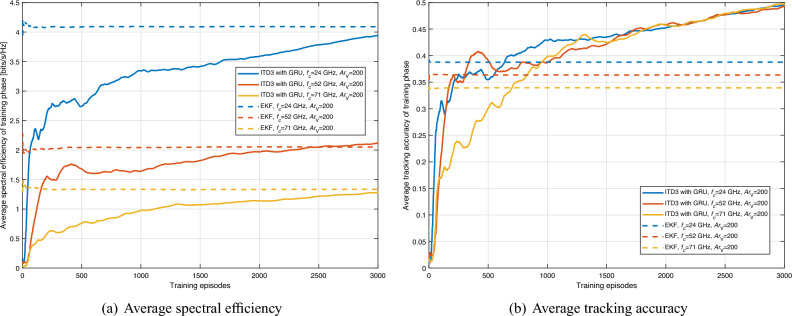
Average spectral efficiency and tracking accuracy of training phase with respect to carrier frequency (ITD3 with GRU).

The average spectral efficiency and tracking accuracy of the training phase are shown in Fig. 5. As we can see, average spectral efficiency and tracking accuracy are improved compared with the IPPO-based method Fig. 4. Also, the ITD3-based method outperforms the baseline method, i.e., the EKF-based method, in tracking accuracy. However, the ITD3-based method has similar spectral efficiency to the EKF-based method after 3000 episodes of training because the EKF-based method uses the narrowest beam to achieve higher beamforming gain. Therefore, for the same tracking accuracy, the EKF-based method has higher spectral efficiency. Despite these improvements, the performance of the ITD3 framework does not yet meet the 5G standards. This is because ITD3 is an off-policy DRL framework, and the policy used for training and testing is not the same. Additionally, the ITD3-based DRL agent adds noise to both actions and policies, resulting in lower performance during training than during testing. The testing performance of the ITD3 framework is shown in Fig. 7.

Upon first glance, the curves in Fig. 5 may seem as though the model hasn’t fully stabilized or converged after 3000 episodes. However, this observation can be attributed to the initial lower performance levels during the early training episodes, which have a pronounced visual impact on the graphical representation. It’s essential to emphasize that the primary objective of the DRL method is the optimization of long-term performance. By the 3000-episode mark, the actual performance of the model has indeed converged, aligning with the model’s long-term optimization goals. The initial setbacks in performance create a visual offset, leading to a perception of non-convergence in the graphical representation. It’s imperative to interpret this within the context of the DRL’s overarching goals and the model’s trajectory across episodes.

**Figure 6:**
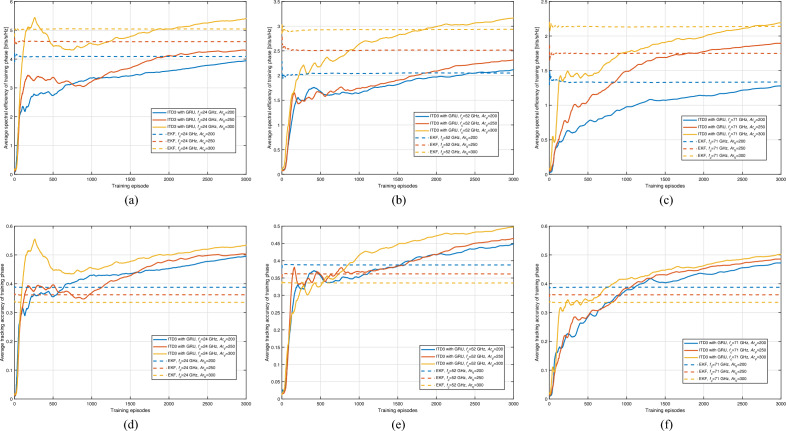
Average spectral efficiency and tracking accuracy of training phase with respect to vehicle density and carrier frequency (ITD3 with GRU).

By the conclusion of 3000 training episodes, evident across Fig. 6, the ITD3 with GRU model manifests a marked maturation in spectral efficiency and tracking accuracy across varied settings. This elongated training phase has granted the model the leeway to thoroughly explore the action and policy spaces, optimizing its response to complex vehicular network dynamics. A predominant factor influencing performance is interference. As vehicle density (i.e., $Ar_{\textrm{V}}$) increases, the inherent challenge of interference intensifies due to multiple simultaneous communications. Yet, the ITD3 with GRU model showcases resilience, attributing to its capabilities of handling complicated scenarios. The model’s adeptness at maneuvering this balance becomes even more commendable when considering the dual role of vehicle density-both as a source of interference and as a factor that reduces communication distances, improving signal power. That is to say, the implications of vehicle density are twofold. On one hand, denser networks foster closer source-destination proximities, enhancing signal power. Conversely, increased density augments interference. The model’s spectral efficiency and tracking accuracy trajectories across episodes, particularly in the post-2000 episodes phase, underline its ability to adapt and harmonize these conflicting dynamics. Furthermore, varying carrier frequencies introduce an added layer of complexity. Different frequencies correspond to distinct path loss profiles even with consistent communication distances. For instance, higher frequencies, such as $f_{\textrm{c}}=71$ GHz, typically experience more significant path loss. However, the model’s performance, especially at the conclusion of 3000 episodes, signifies its versatility in adjusting to these differences. It’s noteworthy that despite these variations in path loss, the model’s spectral efficiency and tracking accuracy remain commendably consistent across the board.

**Figure 7:**
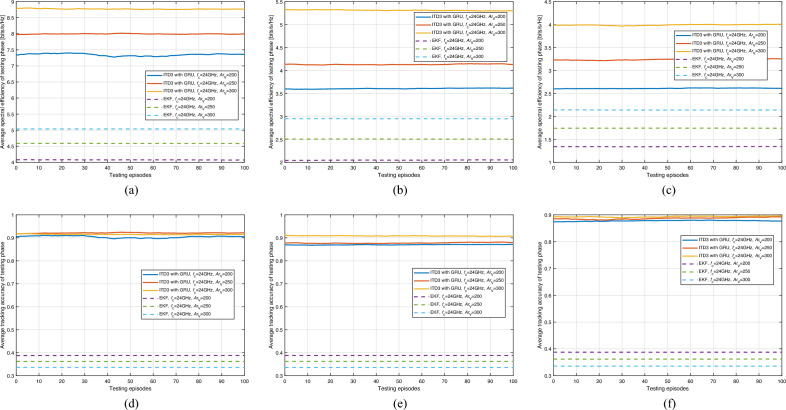
Average spectral efficiency and tracking accuracy of testing phase with respect to vehicle density and carrier frequency (ITD3 with GRU).

The results of the ITD3 framework with GRU during testing are presented in Fig. 7, displaying the average spectral efficiency and tracking accuracy. As demonstrated in the figure, the tracking accuracy exceeds 90%, indicating the capability to manage V2V communication even when the transmission frequency is as high as 71GHz. Also, as we can see from the figures, the average spectral efficiency increases with the density of vehicles. There are possibly two reasons underlying this phenomenon: (1) As the beamwidth narrows, the energy of the beam becomes more concentrated, resulting in lower interference to the surrounding environment from the sidelobes. (2) Under high-frequency transmission conditions (i.e., 5G NR FR2), the signal attenuates rapidly with increasing distance. Thus, as the vehicle density increases, the interference attenuates even more drastically than the signal, primarily because the sources of interference are often at greater distances.

Notably, the testing performance is considerably higher than the training performance, which is attributed to the characteristics of the ITD3 framework. Specifically, DRL methods with continuous action space, such as DDPG and TD3, are designed to introduce noise to action (e.g., ${\textbf{a}}_{i,j}^{m,n}$) during training to encourage adequate exploration of the action space. Consequently, the action during training may not be the optimal one for achieving the best performance due to noise. However, when the fully trained DRL agent is used during testing, the action space does not require further exploration. As such, the action ${\textbf{a}}_{i,j}^{m,n}$ during testing is the most suitable for optimal performance. Additionally, the ITD3-based methods can make vehicle position prediction and dynamic beam pattern selection integrated based on past experiences, while EKF-based methods would require additional optimization algorithms designed for beam pattern selection to achieve the same functionality. These findings suggest that the ITD3 framework with GRU can effectively capture the mobility of VUs and assist in selecting a better codeword, ultimately improving overall performance.

**Figure 8:**
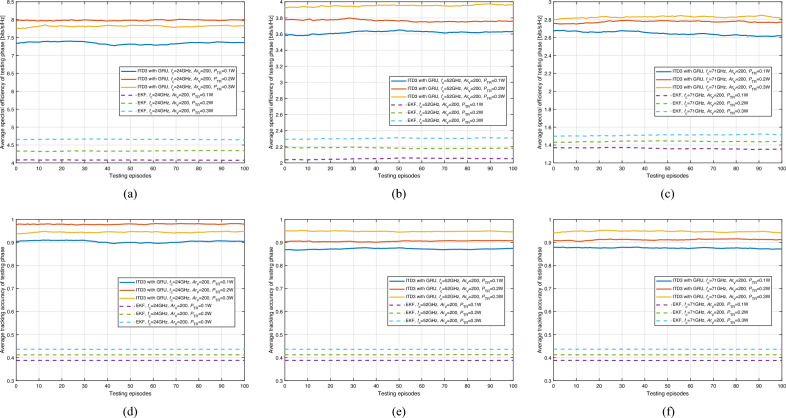
Average spectral efficiency and tracking accuracy of testing phase with respect to transmit power and carrier frequency (ITD3 with GRU).

Figure 8 presents the average spectral efficiency and tracking accuracy during the testing phase concerning transmit power and carrier frequency for the ITD3 with GRU method in comparison with the EKF. The following observations can be made: (1) Across different carrier frequencies, ITD3 with GRU consistently demonstrates a superior spectral efficiency compared to EKF, regardless of the transmit power. This can be observed in sub-figures 8a to c; (2) Sub-figures 8d to f elucidate the tracking accuracy of the two methods. ITD3 with GRU maintains an appreciable tracking accuracy across the board, marginally outperforming EKF in most scenarios. It’s noteworthy to mention that the difference in performance between the two methods becomes more evident at higher transmit powers; (3) For all carrier frequencies, increasing the transmit power from 0.1 to 0.3 W doesn’t lead to significant improvements in spectral efficiency or tracking accuracy for either method. This is primarily because, as $P_{\textrm{TR}}$ increases, not only does the signal strength linearly increase, but the interference strength also rises linearly, with only the noise power remaining unchanged. Under these conditions, performance enhancement mainly results from the increase in the signal-to-noise ratio (SNR). However, this improvement is relatively limited under high SNR conditions.

**Figure 9:**
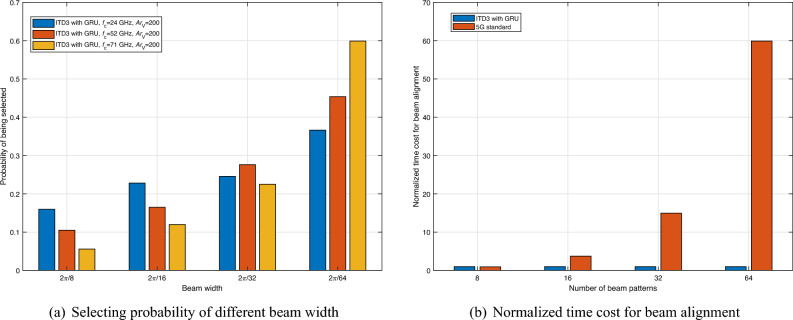
Selecting probability and corresponding normalized time cost for beam alignment.

Figure 9a shows the selection probability of different beam widths. As we can observe, for the lower carrier frequency (24 GHz), the AI agent supported by TD3 with GRU is more likely to select a beam pattern with a wider beam width. When the transmission frequency is 52 GHz or 71 GHz, the AI agent supported by ITD3 with GRU starts to choose narrower beam width, e.g., $2\pi /64 = {1.40625^ \circ }$, to achieve a higher beamforming gain to compensate for the higher path loss. This observation indicates that the ITD3 framework with GRU can successfully help the RSI to determine a more robust beam pattern while considering VU mobility and channel conditions.

On the other hand, due to the variation in the execution time of the same algorithm on different devices, we define the time consumed by the ITD3-based method as one unit of time in Fig. 9b and use normalized time cost to compare the difference in latency between the ITD3-based method and the 5G-based method. As we can observe from Figure 9b, our method outperforms the 5G-based method, even when the number of beam patterns is 8. Furthermore, the tracking latency increases dramatically with the number of beam patterns when the 5G-based method is used but remains stable with our method. This is because our method uses the DRL framework to determine the codeword directly without searching the codebook. Therefore, our method can use more beam patterns to assist beamforming to obtain higher spectral efficiency and beam tracking accuracy.

## Future work

As we progress through our exploration of vehicular networks using DRL framework, a multitude of possibilities beckon for further research. Building on the insights gained from our study, the following areas are earmarked for future exploration:

1. Advanced DRL Models: While TD3 with GRU showcased promising results, the domain of Deep Reinforcement Learning is vast. Other advanced models and algorithms could be explored, potentially leading to improved spectral efficiency and accuracy across different frequencies.
2. Real-world Testbeds: Simulations provide valuable insights, but real-world testbeds introduce unpredictabilities that can significantly influence results. Implementing our methodologies in a real-world setting will provide practical insights, emphasizing areas that simulations might overlook.
3. Integration with Other Technologies: As vehicular networks evolve, their integration with emerging technologies like edge computing, IoT, and B5G/6G becomes inevitable. Future work could explore how these integrations affect the performance of our method, paving the way for more robust and efficient vehicular communication systems.
4. Enhanced Training Strategies: The current study observed a spectral efficiency difference between ITD3/IPPO framework and EKF based on the frequency. Fine-tuning the training strategies, perhaps by integrating other tricks, could extend and optimize the performance of our method to other network conditions.
5. Holistic Network Analysis: Beyond spectral efficiency and tracking accuracy, vehicular networks comprise numerous other performance metrics, such as throughput and reliability. Future studies could adopt a more holistic approach, exploring the comprehensive performance implications of the chosen methodologies.

In conclusion, while our research provides valuable insights into vehicular networks using DRL framework, the journey is far from over. Each avenue mentioned above holds the promise of further optimizing and refining vehicular communication systems, ensuring they are equipped to meet the ever-evolving demands of tomorrow.

## Conclusion

The focus of this paper is on beam management for V2V communications, which presents a challenging task due to various factors, including the short duration of each time slot, high velocity of vehicles, and estimation errors of vehicles’ locations. Additionally, higher transmission frequencies exacerbate the challenges, leading to increased path loss and the need to balance spectral efficiency and tracking accuracy. To address these issues, we propose a DRL-assisted method that accounts for all aspects of vehicles’ mobility and transmission frequency. Our approach involves an analysis of the mobility of vehicles, revealing a high temporal dependency, and the modification of the IPPO framework to the ITD3 framework with GRU. The simulation results demonstrate that the ITD3 framework with GRU outperforms both the IPPO framework and EKF-based method. Specifically, the proposed ITD3 framework achieves high spectral efficiency while maintaining high tracking accuracy and low latency.

## Data Availability

The data supporting this study’s findings are available from the corresponding author, X.G., upon reasonable request.
